# Geospatial model of COVID-19 spreading and vaccination with event Gillespie algorithm

**DOI:** 10.1007/s11071-021-07186-5

**Published:** 2022-01-22

**Authors:** Alexander Temerev, Liudmila Rozanova, Olivia Keiser, Janne Estill

**Affiliations:** Institute of Global Health, University of Geneva, Geneva, Switzerland

**Keywords:** COVID-19, Spatial epidemic modeling, Gillespie algorithm, Contact matrices, Population density

## Abstract

We have developed a mathematical model and stochastic numerical simulation for the transmission of COVID-19 and other similar infectious diseases that accounts for the geographic distribution of population density, detailed down to the level of location of individuals, and age-structured contact rates. Our analytical framework includes a surrogate model optimization process to rapidly fit the parameters of the model to the observed epidemic curves for cases, hospitalizations, and deaths. This toolkit (the model, the simulation code, and the optimizer) is a useful tool for policy makers and epidemic response teams, who can use it to forecast epidemic development scenarios in local settings (at the scale of cities to large countries) and design optimal response strategies. The simulation code also enables spatial visualization, where detailed views of epidemic scenarios are displayed directly on maps of population density. The model and simulation also include the vaccination process, which can be tailored to different levels of efficiency and efficacy of different vaccines. We used the developed framework to generate predictions for the spread of COVID-19 in the canton of Geneva, Switzerland, and validated them by comparing the calculated number of cases and recoveries with data from local seroprevalence studies.

## Introduction

Numerous epidemic modeling and simulation toolkits have been developed or adapted for COVID-19, ranging from educational models to global-scale comprehensive frameworks [1]. Contact network representations are powerful tools that allow us to understand disease transmission in human populations while taking into account the structure of human interactions, mobility, and contact patterns. Previous studies have paid attention to the influence of various network configurations (scale-free, random, small-world) [2–4] on the spreading rate and value of the epidemic threshold. Some models also work with population mobility data, which is especially important in the context of imposing movement restrictions to reduce transmission [1, 5].

To improve the accuracy of epidemic models, it is necessary to account for population heterogeneity, e.g., in terms of age, social groups, and mobility patterns, as well as the geographic clustering of infection spread that may result from higher contact rates in locations with higher population density. Examples of models with a detailed representation of these factors include the age-structured household model of Pellis et al. [6] and the two-level (global and local) mixing model proposed by Ball et al. [7] which has further been expanded to account for network structure [8]. However, these models do not include distance metrics or account for differences in population density. In the context of analyzing and predicting the course of the COVID-19 epidemic, the impact of various epidemic control and containment measures needs to be thoroughly examined. [9].

### Motivation

The importance of the spatial component in epidemic systems is increasingly recognized. When models are used to evaluate spatially heterogeneous interventions, they must be able to represent the location of hosts and the spatial pattern of transmission in sufficient detail. The inclusion of the spatial component in epidemic models has been considered a complex technical add-on that should only be considered when absolutely necessary [10]. However, as software and methods improve and spatial data at the individual level become increasingly available, we can expect the spatial component to play an increasingly important role in infectious disease modeling.

## Methods

### General assumptions

We use a standard representation of the population as a network consisting of *N* nodes (individuals) $\{i\}$ and a set of links $\{e_{ij}\}$, representing a contact between each node *i* and *j*. We assume that the number of individuals in the network remains constant during the simulation [2]. In our case, the network is weighted (all links $e_{ij}$ have a weight depending on the spatial distance between the nodes *i* and *j*) and complete (i.e., a link $e_{ij}$ exists for any pair of nodes *i* and *j*).

We represent the infection process in the network with a stochastic SEIR model, so that all nodes are at any time point in one of the four states: *S* (susceptible), *E* (exposed in the latent period), *I* (infectious), or *R* (recovered/removed).

The stochastic infection process $S\rightarrow E$ occurs as a result of contact between a susceptible ($i\in S$) and an infectious ($j \in I$) individual. Two other random processes occur in the network in parallel: the transition of individuals from the exposed to the infectious state $E \rightarrow I$ and the removal process $I \rightarrow R$.

The model assumes two types of contact events, which we call “local” and “global” contacts and distinguish on the basis of the Euclidean distance $m_{ij}$, with some radius *r* as the cutoff distance (which is the same for all individuals). Thus, we distinguish between contacts that take place near an individual’s residence, and contacts that take place during travel over longer distances.

Local contacts are determined by a cutoff *r* on the distance $m_{ij}$. This cutoff is the same for all nodes in the network. Global spread is modeled as a separate process in which each infectious individual can spontaneously transmit the infection to a randomly selected target from the entire population. The process is automatically normalized for population density, as the target is more likely to be located in densely populated areas.

In our model, the following parameters affect the contact probability between two nodes:

1. The assignment of nodes to one of 16 age groups. The frequency of contacts between age groups is given as an asymmetric 16x16 matrix $A = \{a_{ij}\}$ [Appendix A].
2. The distance between nodes. Since only relative distances matter in our model, we use the Euclidean distance between locations on the map. We set a fixed cutoff distance to distinguish between local and global contacts.
3. The population density in a given area. The contact probability for global contacts depends directly on the population density in the area of the contacting nodes—this is achieved by rejection sampling of global contacts over the entire simulated region.

### Epidemic model

We represent the stochastic SEIR model as a continuous time three-dimensional Markov chain $X=\{(S(t),E(t),I(t)):t\ge 0 \}$ that tracks the number of susceptible, exposed and infectious individuals at each time point. The number of removed individuals can be calculated as $R(t)=N-S(t)-E(t)-I(t)$, where *N* is the total number of individuals, assumed to be constant during the simulation.

The epidemic starts with $n_0$ infectious individuals; the rest of the population is assumed to consist of fully susceptible individuals, i.e., the initial state of *X* is $(S(0),E(0),I(0))=(N - n_0,0,n_0)$. At each time point, the state space of *X* is described by changing the state of the individuals according to the rules listed in Table 1. All other types of contacts do not change the state of the system.

We use Poisson processes for link activation. When an infection event occurs at a node $i\in I$, we first randomly select a node *j* within Euclidean distance $m_{ij}$ smaller than *r*, and activate the link $e_{ij}$ with probability that depends only on the age-contact structure of the population: $p_{ij} = a_{ij}/K$, where *K* is the normalization factor: the total contact rate is summed for all age groups. If *j* is in the susceptible state, the disease is transmitted with probability $\beta ^L_{ij}=\beta p_{ij}$, where $\beta$ is the transmission rate and the susceptible node becomes infected (exposed).

For global contacts, we select a random node *j* over the entire simulated geographic region with rejection sampling, such that the target node is selected according to the population density distribution. This takes into account all non-local contacts (chance encounters, travel, etc.) and allows us to specify their relative proportion with a single parameter: the frequency of global contacts relative to local contacts. In this case, the disease is transmitted with probability $\beta _{ij}^G =\beta p_{ij}d_j/D$, where $d_j$ is the density of the local area of global contact *j* and *D* is a normalizing constant.

An infected node remains in the exposed state for a time interval (exponentially distributed) calculated from the mean rate $\epsilon$, after which it transitions to an infectious state where it can infect other nodes. The transition to the recovered state follows another Poisson process with exponentially distributed time intervals calculated from the rate $\gamma$. A recovered node neither infects other nodes nor is infected by other nodes.

**Table 1:** Transition rates between Markov chain states

Transition	Type	Rate	State change
$S \rightarrow E$	Local/global contagion	$\beta _{i}$	$(s_t-1,e_t+1,i_t)$
$E \rightarrow I$	Spontaneous	$\epsilon _{i}$	$(s_t,e_t-1,i_t+1)$
$I \rightarrow R$	Spontaneous	$\gamma _{i}$	$(s_t,e_t,i_t-1)$

### Simulation algorithm

To simulate the infection process, we developed and applied a new variant of the event-modulated Gillespie algorithm. Our implementation supports multiple epidemic control regimes and arbitrary functional forms and distributions of epidemic parameters. It can be used to validate theoretical models and is particularly suitable for simulating epidemic dynamics in large regions such as entire countries.

The unmodified Gillespie algorithm is described as follows. Consider *N* Poisson processes with rates $\uplambda _i$, $i\in [1,N]$ running in parallel. We denote the density of the event rate for the *i*th process by $\rho _i(\uplambda _i)$. The renewal process is completely characterized by the probability density of inter-event times. For the Poisson process with probability density of event rate $\rho _i(\uplambda _i)$, we have

$$\begin{aligned} \psi _i(\tau ) = \int _0^{\infty }\rho _i(\uplambda _i)e^{-\uplambda _i \tau }d\uplambda . \end{aligned}$$

A Poisson process with rate $\uplambda _0$, i.e., $\psi _i(\tau ) = \uplambda _0 e^{-\uplambda _0 \tau }$ is generated by $\rho _i(\uplambda _i) = \delta (\uplambda _i - \uplambda _0)$, where $\delta$ is the delta function.

In the unmodified Gillespie algorithm applied to epidemic spreading, all possible state transitions for nodes ($S\rightarrow E$, $E\rightarrow I$, $I\rightarrow R$) are Poisson processes with rates *i*. Multiple simultaneous Poisson processes can be combined into a single global Poisson process with the rate $\varLambda = \sum _{i=1}^N\uplambda i$, where the time intervals between events are distributed exponentially with the geometric mean $1/\varLambda$, and for each event of this process, we need to calculate what exactly that event is, and where it occurred. (To do this, we need to keep track of all susceptible nodes that can be infected immediately, all exposed nodes that can transition to the infectious state, and all infectious nodes that can be recovered and sample the probabilities of these transitions accordingly.) The original Gillespie algorithm was first developed to track chemical reactions and later adapted to model epidemic processes in well-mixed populations where the only important output is the number of entities in each state (e.g., molecules in chemical models, individuals in epidemic models). However, in network and geospatial models, it is important to know which node exactly makes the transition, leading to computational difficulties.

In our event-modulated Gillespie algorithm, we cut off all unproductive (not leading to a state change) activations, and use a global event queue (a priority queue, i.e., all events are always sorted by time) to automatically place all actual state transitions at the appropriate time points in the future. Therefore, we no longer need to merge all event rates *i* into the global rate $\varLambda$ but instead work directly with the SEIR parameters $\beta$, $\epsilon$, and $\gamma$, using them as the rates of the corresponding Poisson processes and generating exponentially distributed time intervals between events.

When a node becomes infectious (either from the exposed state after the incubation period or because it is one of the original infected patients), the infection sequence begins. First, we sample the time interval until removal (recovery or death) as $-\ln (U)/\gamma$, where *U* is a random value sampled from the uniform distribution [0.1], and place a removal event in the priority queue. We then generate exposure events and their time offsets, which continue as long as the sum of the time intervals between these events is less than the time between the occurrence of the infection and the removal event. The exposure process is described as follows.

We first determine whether the exposure event will be local or global, the probability of a global transmission being *q*, and the probability of a local transmission $1-q$. We then sample an age group for the target node from the discrete distribution taken from a row of the contact matrix corresponding to the source node’s age group. If the exposed contact is local, we select randomly a node *j* that is located within the Euclidean distance $m\le r$ from the index node *i* and has an age group equal to the age group we sampled in the previous step. If the exposed contact is global, we select randomly any node *j* over the entire simulated geographical region using rejection sampling (by generating random uniformly sampled Euclidean coordinates with 10m precision until we have a location that contains a node from the chosen age group), so that the target node is selected according to the population density distribution. Then, we sample the time offset for this exposure event as $-ln(U)/\beta$. If the time of the exposure is still less than the time of the recovery, we place the event in the queue and move on to the next exposure event . If the exposure event is already beyond the removal time, we cancel the last exposure event and stop the process.

The continuous time evolution of stochastic processes with known transition rates (including epidemiological applications) can be numerically simulated with the family of Gillespie algorithms, which are statistically accurate. For our needs, we used a particular variation: the event-modulated Gillespie algorithm [11].

The algorithm itself is outlined in (Algorithm 1), adapted from István Z. Kiss et al. [12]. Its core capabilities (infection spreading depending on contact matrices and geographical location of individuals) are abstracted into the find_contact function (line 17).
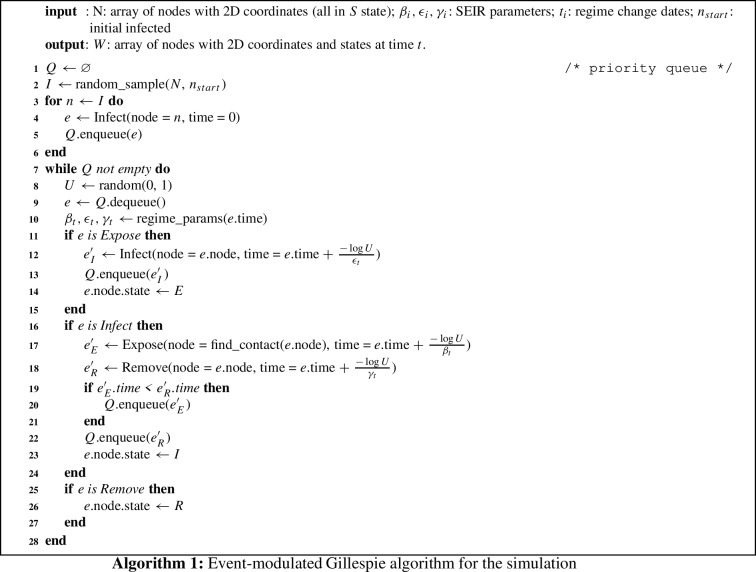


### Vaccination process

Vaccinations have been modeled as a separate process with piecewise linear intensity, running in parallel with epidemic processes. Vaccination capacities were taken from the vaccination statistics dataset for Geneva provided by the Swiss Federal Office of Public Health FOPH and set at 1000, 5000, and 8000 daily vaccinations (from 31 December 2020, 1 May 2021, and 1 June 2021) as a proxy to represent actual vaccine deliveries and administration. Multiple doses of vaccine are supported (set to 2), with a set interval between doses (set to 21 days, as for Pfizer-BioNTech’s COVID-19 vaccine).

Since the simulator works with explicit individuals placed in the 2D environment, we assign a susceptibility value to each individual, initially set to 1.0. Susceptibility functions like a coefficient applied to the probability of infection of the target individual (or equivalently, as a multiplier for the time between infections). Each dose of vaccine administered multiplies the susceptibility coefficient for the target individual by a predetermined number. (Currently set at 0.7—so after a single dose, the individual is $70\%$ less susceptible, and after a second dose, $91\%$ less susceptible, compared to baseline—this represents the efficiency of Pfizer-BioNTech’s vaccine.)

We do not explicitly model the difference between vaccine efficiency and vaccine effectiveness—we assume that there is sterilizing immunity and that the spread of virus is stopped if a vaccinated individual has not become infected. (The probability of this is $91\%$ lower after two vaccine doses.) In any case, we consider only the last effect: the reduction in hospitalizations, and fit the simulation accordingly. Currently, the lower efficacy of the vaccines for the new variants is proxied by a correspondingly higher infectiousness.

**Fig. 1:**
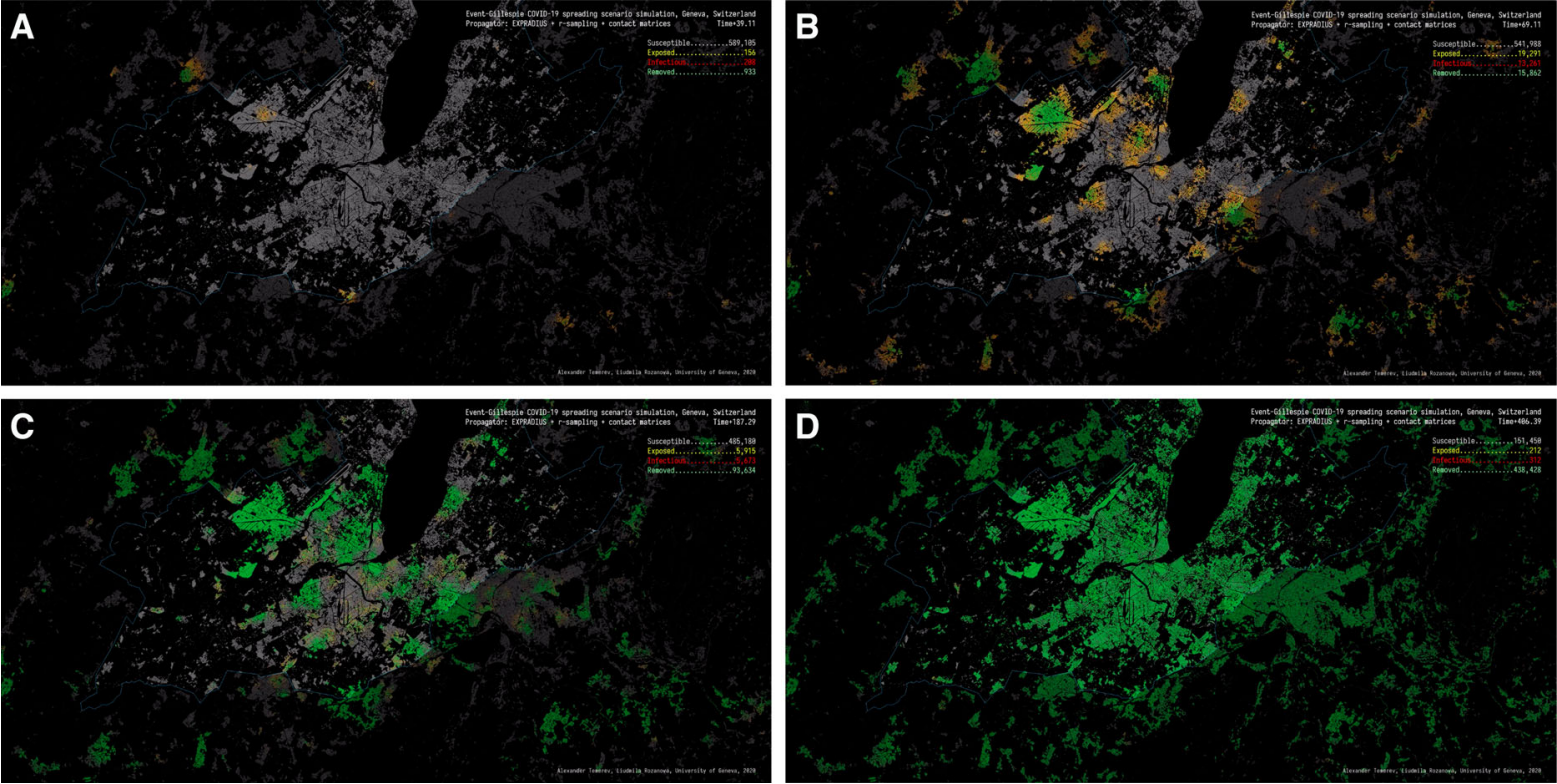
Simulation state for the canton of Geneva, Switzerland: **A** 3 March 2020; **B** 2 April 2020; **C** 6 October 2020; **D** 6 October 2021. Each colored pixel corresponds to a state of a single individual. Grey pixels are the susceptible (S) individuals, yellow are exposed (E), red are infectious (I), and green are removed (R)

Simulation supports limiting vaccine availability to selected age groups only. In our simulations, we made vaccines available to people aged 65 and older (starting 31 December 2020), 45 and older (starting 1 May 2021), and 18 and older (starting 1 June 2021).

### Simulation running

The main simulation code is written in C++ (C++17) and uses the GDAL [13] library for geospatial transformations and manipulations, and the nanoflann [14] k-d tree implementation for radius queries. The population density map from the Global Human Settlement Layer dataset is loaded as a 2D array, with areas inside and outside the border marked separately. The state of the simulation is exported to an output log on each day of the simulation as current SEIR counts, and as a PNG file representing the current state of the population on the density map, where susceptible individuals are represented by grey pixels, exposed by orange pixels, infected by red pixels, and removed by green pixels. Localized clusters of infection in the simulation as well as the direction of infection spread are thus clearly visible.

A single simulation run for the population of the Canton of Geneva (N=499,480) takes on average 35 minutes. For N=10,000,000, the average run time for a single run was about 120 minutes. The simulation has a high degree of stochasticity, so the time required to complete it varies considerably. A single simulation run consumes about 6 GB RAM for N=10,000,000. All simulation runs are independent and therefore trivially parallelizable. We performed all simulations using the Yggdrasil HPC cluster at the University of Geneva, managed with SLURM.

We run a series of 500 simulations, each simulation starting with the same initial condition where a set of randomly selected nodes is infected and all other $N-n_0$ nodes are susceptible (in this particular configuration $n_0 = 2$). We measure the percentage of recovered nodes at the end of the simulation (final size) and use the mean estimator from Seaborn [15] with $\pm 1\sigma$ interval to conveniently display the data from multiple simulations. The number of nodes in different states can be aggregated and converted into different indicators to be displayed as stochastic epidemic curves. Figure 1 shows the internal state of the simulation in the population density map, where each pixel corresponds to a single individual, and the epidemic states are shown in different colors.

The distance matrix *M* is constructed using information about population density for each simulated region, obtained from the ESM2015 dataset [16]. The age-dependent contact matrix *A* comes from the work of Kiesha Prem et al. [17], which extended the results of the POLYMOD project [18] to 152 countries.

### Surrogate model optimization

To provide the initial epidemic parameters for the full simulation, we use surrogate model optimization. We fit a stochastic SEIR model that assumes a homogeneous contact network to match observed data on COVID-19 cases in Switzerland provided by ETH Zurich. [19].

We wrote an adapter function to run the surrogate model and export the simulation results, and compared the results to the actual observed number of hospitalizations for the selected geographic region. Then, we wrote a nonlinear least squares optimization routine curve_fit from the scikit-optimize Python package, which works with arbitrary model functions. We use the trusted region reflected optimization algorithm, which is one of the available algorithms that is general and robust enough to be used in bounded parameters search.

After fitting, the obtained parameter values are used as inputs for the simulation of the main model, leaving only the local infection distance threshold *r* and the fraction of global contacts in all contacts as free parameters.

In the first stage of the fitting procedure, we fix the values $\epsilon$ and $\gamma$ of the SEIR model (inverse incubation time and inverse infectiousness interval, since they do not change) as *X* and *Y*, respectively, and assume that $\beta$ ( contagion rate) changes gradually over time due to the different epidemic control regimes. We assumed that the first infections occurred between January 15 and February 25. The first case was officially registered in the canton of Geneva on 25 February, but the infection had probably already been spreading at that time.

The following parameters were fitted:

1. $\beta _k$—infection spreading rates for each time interval ($\beta _0$ bounded from initial estimations of $R_0$);
2. $t_0$—the start date of the epidemics

The bounds are shown in Table 2.

The fitting routine consists of solving the optimization problem of finding the minimum of the function:

$$\begin{aligned} F(\theta )=\sum _{i=1}^N (f_i(\theta )^2), \end{aligned}$$

where $\theta = (\theta _1,...,\theta _r)$ is a set of parameters we want to estimate, *N* is the number of available data points, $f_i(\theta )$ is the *i*-th component of the vector of residuals. (In this particular simulation, we are fitting for the number of hospitalizations since this is the most objective and readily available statistic, assuming that the proportion of hospitalized patients always remains the same for each age group, at least within the region under consideration.)

Given a model function $m(t;\theta )$ and some data points $D=\{(t_I,D_I|i=1, .., N\}$, we defined the vector of residuals as the difference between the model prediction and the data, i.e., $F_i(\theta )=m(t_i;\theta ) -d_i$.

**Table 2:** Fixed and free parameters to be fitted in the surrogate model

Name	Description	Fixed value or search bounds	Fitted values
$\beta _k$	Infection rates (for different regimes)	0.01–2.5	1.0, 0.7, 0.09, 0.2, 0.2, 0.34, 0.59, 0.3, 0.1, 0.3, 0.15, 0.34, 0.41
$\epsilon$	Incubation rate	0.2	N/A
$\gamma$	Removal rate	0.16	N/A
$t_0$	Epidemic start date	15 January 2020–25 February 2020	24 January 2020
$t_k$	Epidemic regimes change times, days from the start of the epidemic	[0], 40, 51, 110, 138, 166, 225, 265, 282, 301, 357, 370, 463.	N/A

We compared the simulation results with real observations from the clinical dataset COVID-19 provided by the FOPH [19]. The epidemic parameters determined in the fitting step were passed to the main simulation code, which was then run 500 times independently. The initial mean contact radius *r* for the local transmission mode was set to 500 meters, as adapted from [20], and modified over time between 50 m and 500 m according to changes in mobility. The proportions of global contacts were taken from the same study and ranged from $0.01\%$ to $1.00\%$, according to the mobility data. For comparison purposes, we used the number of hospitalizations as a reference value. We considered hospitalizations as a subclass of the *R* (removed) compartment because hospitalized patients do not typically infect other individuals. The percentages of infected persons in each age group assumed to be hospitalized are shown in Fig. 3. We also compared the cumulative percentage of recovered patients in the simulation with figures from seroprevalence studies conducted in the canton of Geneva [21].

**Fig. 2:**
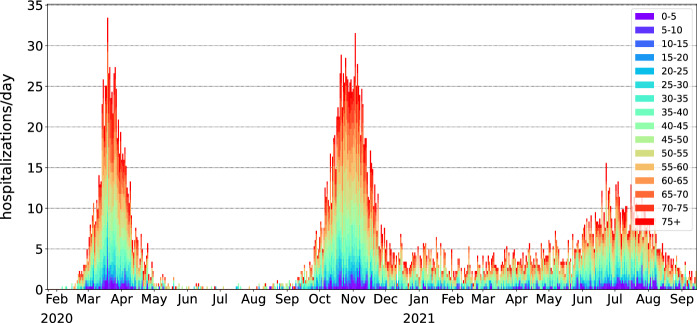
Simulation: daily hospitalizations in the canton of Geneva colored by age group

**Fig. 3:**
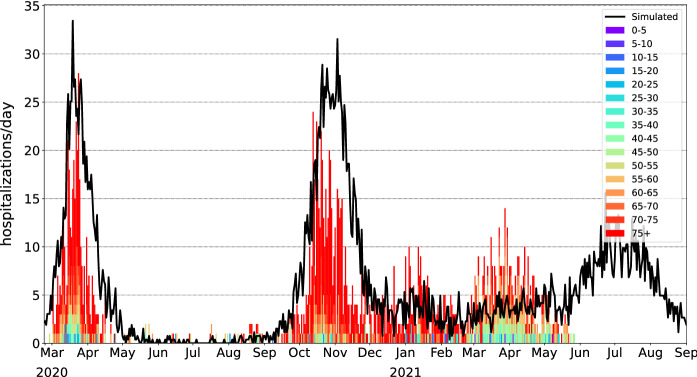
Daily hospitalizations in the canton of Geneva by age groups (the colored area chart), compared to the simulation results (the black curve)

**Fig. 4:**
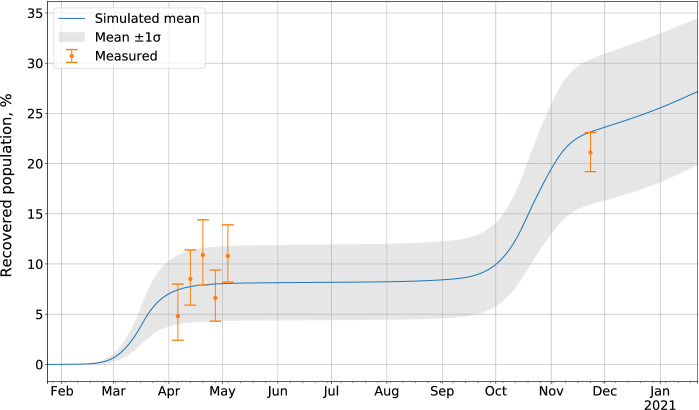
Percentage of recovered population in the canton of Geneva, aggregated data from 100 simulation experiments with $\pm 1\sigma$ error bands, compared to measured seroprevalence estimates. [21]

The epidemic parameters obtained in the surrogate model are shown in Table 2. The simulation reproduced the first epidemic wave of COVID-19 that occurred in March–April 2020 (Figs. 2 and 3). The simulated number of recovered cases is consistent with seroprevalence measurements conducted in the canton of Geneva in April–May 2020 and November–December 2020 (Fig. 4).

## Results

The set of epidemic parameters obtained in the surrogate model is shown in Table 2. The simulation reproduced the initial epidemic wave of COVID-19 that happened in March–April 2020, as well as subsequent periods of rising numbers of cases and hospitalizations (Figs. 1, 2 and 3). Even with the vaccination program in place, the simulation predicts an increase in number of cases in July–August 2021, as new variants of concern are up to 100% more infectious compared to the baseline variant [22]. The effect of vaccinations, however, accumulates steadily leading to the predicted fall in the number of new hospitalizations in mid-September 2021. At the time of this writing, these predictions need to be validated by further observations.

Importantly, the simulated numbers of recovered cases is consistent with seroprevalence measurements performed in the canton of Geneva in April–May 2020 and November–December 2020 [21] (Fig. 4).

## Results and discussion

We have developed a flexible network simulation model for COVID-19 and successfully tested it with data from the canton of Geneva, Switzerland. Our system allows us not only to track and predict the course of the epidemic, but also to simulate and evaluate different measures put in place to contain the spread of the infection, such as restricting mobility and partial or complete lockdown. The simulation software also makes it possible to model the effectiveness of vaccination and decide which populations (based on age or location) should be prioritized for vaccination.

**Table 3:** A contact matrix for 16 age groups in Switzerland [17]

Age	0-5	5-10	10-15	15-20	20-25	25-30	30-35	35-40	40-45	45-50	50-55	55-60	60-65	65-70	70-75	75+
0-5	1.35	0.56	0.25	0.14	0.21	0.39	0.69	0.73	0.49	0.22	0.22	0.18	0.14	0.13	0.07	0.04
5-10	0.48	5.67	0.89	0.22	0.15	0.33	0.62	0.80	0.88	0.37	0.21	0.16	0.14	0.12	0.05	0.05
10-15	0.18	1.74	9.11	0.81	0.29	0.25	0.43	0.68	1.11	0.64	0.34	0.15	0.09	0.10	0.07	0.07
15-20	0.09	0.29	3.11	11.68	1.50	0.77	0.64	0.79	1.09	1.20	0.67	0.24	0.08	0.06	0.03	0.03
20-25	0.13	0.17	0.31	2.35	3.95	1.77	1.23	1.15	1.01	1.32	0.98	0.49	0.12	0.06	0.06	0.06
25-30	0.35	0.24	0.24	0.89	2.07	3.66	1.91	1.52	1.36	1.17	1.21	0.68	0.21	0.08	0.04	0.04
30-35	0.59	0.80	0.64	0.52	1.06	1.82	3.13	1.98	1.58	1.26	0.96	0.67	0.29	0.14	0.06	0.06
35-40	0.61	0.95	0.77	0.74	0.77	1.45	1.81	3.14	2.22	1.46	1.06	0.53	0.29	0.21	0.11	0.05
40-45	0.35	0.80	1.03	1.20	0.97	1.34	1.73	1.99	3.19	1.89	1.34	0.47	0.22	0.16	0.11	0.06
45-50	0.19	0.58	0.79	1.86	1.00	1.12	1.39	1.59	1.79	2.57	1.37	0.60	0.20	0.13	0.11	0.12
50-55	0.17	0.64	1.11	1.53	1.15	1.48	1.38	1.38	1.91	2.15	2.37	1.00	0.30	0.16	0.10	0.12
55-60	0.30	0.66	0.79	0.94	0.75	1.27	1.35	1.07	1.29	1.13	1.33	1.68	0.52	0.27	0.12	0.11
60-65	0.31	0.33	0.26	0.44	0.38	0.61	0.82	0.79	0.63	0.56	0.51	0.71	1.23	0.49	0.26	0.12
65-70	0.23	0.35	0.28	0.17	0.27	0.42	0.73	0.68	0.65	0.43	0.44	0.58	0.63	1.29	0.33	0.17
70-75	0.10	0.27	0.31	0.31	0.17	0.29	0.33	0.53	0.66	0.52	0.41	0.35	0.67	0.72	1.00	0.32
75+	0.20	0.27	0.40	0.33	0.15	0.18	0.33	0.38	0.48	0.57	0.56	0.35	0.27	0.40	0.33	0.56

Because organizations responding to epidemics often have limited resources, optimal resource allocation is critical to develop the most effective interventions. This includes identifying the most vulnerable populations groups, identifying epidemic hotspots, and determining the optimal timing and locations for introducing non-pharmaceutical interventions. Models without a spatial dimension are limited in their ability to answer some questions about intervention effectiveness and resource allocation [8, 10]. Our framework uses age-based contact matrices to account for differences in social networks and contact patterns across age groups. It uses detailed population density maps to predict and visualize geographic locations of epidemic hotspots, and it uses a robust optimization framework to adapt to real-world observations and medical statistics available for the target setting, allowing operators to choose optimal response options.

Our approach also has some limitations. The full representation of the population at the level of individuals is computationally much more expensive than traditional compartmental models. Although the computational resources required are still modest compared to many computational physics problems, this makes continuous experimentation and parameter optimization time-consuming and often impractical. Our approach requires some non-standard parameters that are difficult to calculate from observed data (such as the fraction of “locally” transmitted infections).

The addition of some new features could further improve the simulation and the underlying model. These include accounting for seasonality, dividing the geographic representation into zones (e.g., commercial, residential, business, or recreational areas with corresponding differences in mobility patterns), or accounting for travel across the outer boundaries of the simulated environment (which could be represented as spontaneously generated new infection events). However, the addition of these features risks taking the model far beyond analytical tractability and reducing it to an empirical numerical simulation, limiting our ability to generate new theoretical results. Further research is needed to prioritize the approaches with the greatest robustness and practical utility.

Although this model is more computationally intensive than conventional compartmental models and requires some unconventional parameters, our approach can make the simulation results more reliable. This model provides an efficient way to account for age and geographic structure and mobility without resorting to even more computationally intensive direct simulations of people movement.

## Data Availability

The original datasets are available at their respective URLs; the prepared datasets for the simulation (population density map for the canton of Geneva, population pyramid data, etc.) are available within the project’s Gitlab repository [23].

## Appendix A

See Table 3.

## Declaration

### Code availability

The code for the simulation and postprocessing scripts is available at the project’s repository [23].
